# Label-free classification of neurons and glia in neural stem cell cultures using a hyperspectral imaging microscopy combined with machine learning

**DOI:** 10.1038/s41598-018-37241-y

**Published:** 2019-01-24

**Authors:** Hiroshi Ogi, Sanzo Moriwaki, Masahiko Kokubo, Yuichiro Hikida, Kyoko Itoh

**Affiliations:** 1 Department of Interdisciplinary Research & Development, Graduate School of Medical Science, Kyoto Prefectural University of Medicine (KPUM), Kyoto, Japan; 2 Department of Pathology and Applied Neurobiology, Graduate School of Medical Science, Kyoto Prefectural University of Medicine, Kyoto, Japan; 3 SCREEN Holdings Co., Ltd. (SCREEN), Kyoto, Japan

## Abstract

Due to a growing demand for a viable label-free observation method in the biomedical field, many techniques, such as quantitative phase imaging and Raman spectroscopy, have been studied, and a complementary approach, hyperspectral imaging, has also been introduced. We developed a high-speed hyperspectral imaging microscopy imaging method with commercially available apparatus, employing a liquid crystal tunable bandpass filter combined with a pixel-wise machine learning classification. Next, we evaluated the feasibility of the application of this method for stem cell research utilizing neural stem cells. Employing this microscopy method, with a 562 × 562 μm^2^ field of view, 2048 × 2048 pixel resolution images containing 63 wavelength pixel-wise spectra could be obtained in 30 seconds. The neural stem cells were differentiated into neurons and astroglia (glia), and a four-class cell classification evaluation (including neuronal cell body, glial cell body, process and extracellular region) was conducted under co-cultured conditions. As a result, an average of 88% of the objects of interest were correctly classified, with an average precision of 94%, and more than 99% of the extracellular pixels were correctly segregated. These results indicated that the proposed hyperspectral imaging microscopy is feasible as a label-free observation method for stem cell research.

## Introduction

These days in the fields of life science, biology and medicine, there has been a growing demand for label-free observation methods that allow for the assessment of biological materials repeatedly without any toxic effects and without the use of dyes or tags. One of the areas where this demand has been prominent is the field of pluripotent (or multipotent) cell research. Pluripotent cells, for example, induced pluripotent stem cells^1^ (iPSCs), embryonic stem cells^2,3^ (ESCs) and neural stem cells^4^ (NSCs), have regenerative abilities and the capability to differentiate into multiple types of cells. Given their unique abilities in regeneration and pluripotency, regenerative and reparative therapies and cell-based therapies using these pluripotent cells have been intensively studied for a decade or more^5,6^. In addition to their direct use in therapy, they can also serve as powerful research tools for understanding the molecular mechanisms involved in intractable diseases by building precise human models, for example, in neurodegenerative disorder Parkinson’s disease^7^, Huntington’s disease^8^, amyotrophic lateral sclerosis^9^, Alzheimer’s disease^10^ and fetal developmental defect myelomeningocele^11^. While pluripotent cells have potential roles to play, as mentioned above, and due to their regenerative ability and pluripotency, it is necessary to carefully control and monitor their differentiation processes repeatedly in order to achieve successful results. Under different culture environments, pluripotent cells can differentiate into different types of cells^1^, and there is a dangerous possibility for tumor formation associated with residual undifferentiated cells used in autologous therapy^12^.

Among these label-free observation methods, quantitative phase imaging (QPI) and Raman spectroscopy (RS) have been intensively studied recently. QPI employs the principle of interferometry to measure the optical field, obtaining amplitude and phase information similarly to conventional phase contrast microscopy (PCM), but quantitatively^13,14^, and it has been successfully utilized for monitoring the physiological states of living cells^15^. RS measures the collection of vibrational spectra that are scattered during the Raman scattering process, and the spectrum can be uniquely mapped to molecular compounds. RS techniques, including imaging, have been employed for pharmaceutical applications^16^ and for monitoring live cell physiology^17–19^. Although there are many promising reports on QPI and RS, both techniques have disadvantages for practical applications in stem cell research. QPI requires homogeneous reference fields for accurate phase measurements^13^, which are sometimes difficult to obtain, and in RS, because the Raman scattering is a weak effect, signals are highly sensitive to the observation setup^13^ employed and measurements are time consuming, for example, it takes around 5 min to get a 235 × 111 pixel image^17^.

In recent years, yet another technique, hyperspectral imaging (HSI), which may be applicable to label-free noninvasive observation and complementary to QPI and RS, was introduced in the life science field^20^. A hyperspectral image can provide a complete spectrum of the sample at each pixel together with morphological information. Several kinds of HSI have been proposed including reflectance microscopy^20^, micro-Raman spectroscopy^21,22^ and multispectral fluorescence microscopy^23^. To date, in addition to experiments with stained materials^24^, several fundamental studies reported on the potential of HSI for label-free discrimination of living cells. Bertani F.R. and his colleagues demonstrated that melanoma cells were discriminated from keratinocytes using hyperspectral confocal reflectance microscopy^25^. They also reported semi-automatic classification of macrophagic polarization using the same HSI microscopy^26^. In previous studies, researchers utilized built-from-scratch high-power Laser Scanning Confocal Microscopy (LSCM), which had some photo-damage effect risks and took a long time to obtain data (more than 6 min to get a 512 × 512 pixel, 1 × 1 mm^2^ image, at an acquisition rate of 400 Hz)^26^. Furthermore, only spectral information was used to discriminate cells. In view of the current circumstances regarding HSI, this study was conducted to investigate the feasibility of using the HSI method for rapid label-free observation in stem cell research, employing commercially available apparatus combined with machine learning techniques, utilizing both spectrum and morphological features to identify cells.

## Results

### HSI microscopy

**Figure 1:**
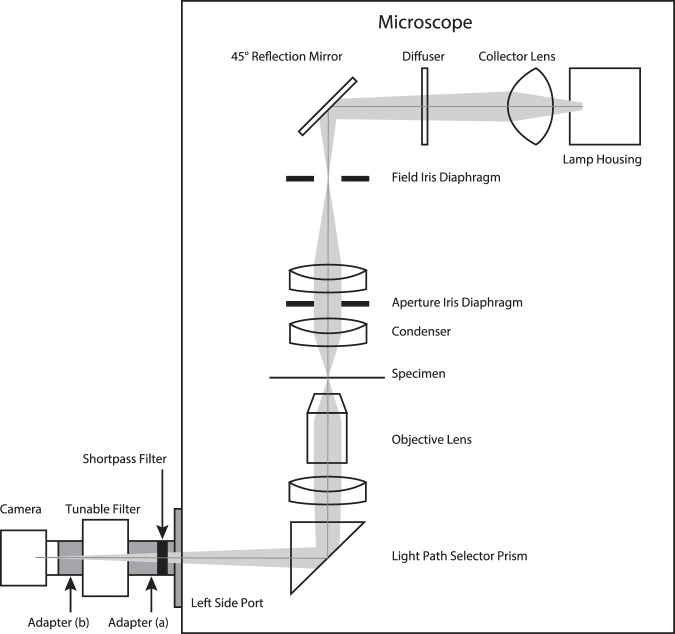
HSI microscopy. The tunable bandpass filter and camera were connected to the port on the left side of the microscope with adapters (**a**,**b**). A shortpass filter was installed in the adapter (**a**), to cut long wavelength light (the cut-off wavelength was 750 nm).

Figure 1 shows a diagram of the HSI microscopy used in this study. The tunable bandpass filter and camera were connected to the port of the microscope on the left side, with two adapters in front of and behind the tunable bandpass filter. In the adapter in front of the tunable bandpass filter (Fig. 1 Adapter (a)), a shortpass filter was installed to cut the long wavelength light.

The correction collar of the objective lens was set to a minus position in order to achieve the finest focus (see Supplementary Figs S1 and S2 for details), and the iris aperture diaphragm was set to a minimum to obtain the largest contrast differences between the cells and the external regions (with this setting, the numerical aperture of the illumination was 0.063). The lateral resolution of this optical setup was 0.820 μm at the 450-nm wavelength, 1.002 μm at the 550-nm wavelength and 1.184 μm at the 650-nm wavelength with a minimum iris aperture diaphragm setting (calculated by Hopkins’ equation^27,28^).

Employing 5 illumination images acquired during a period of 2 hours, we evaluated spatial and spectral stability of the optical setup at representative 450-nm, 550-nm and 650-nm wavelengths (the evaluation included the illumination, the microscope, the bandpass filter and the camera). More than 90% pixels showed within 5% fluctuation of the intensity (5% of the mean intensity at each wavelength) during the 2 hours at all three wavelengths. Overall, 75% of the pixels showed within 4% fluctuation, and 50% of the pixels showed within 3% fluctuation at all three wavelengths.

The first of the five illumination images was evaluated for spatial stability, and 14.1%, 3.5% and 4.1% (percent of the mean intensity at each wavelength) non-uniformity of intensity in the field of view were observed (at 450-nm, 550-nm and 650-nm wavelengths, respectively). The patterns of the non-uniformity differed among the wavelengths, especially at the 450-nm wavelength (Supplementary Fig. S3). This non-uniformity was canceled by the flat-field correction for observation and cell classification employed in this study.

### Observation of NSCs

**Figure 2:**
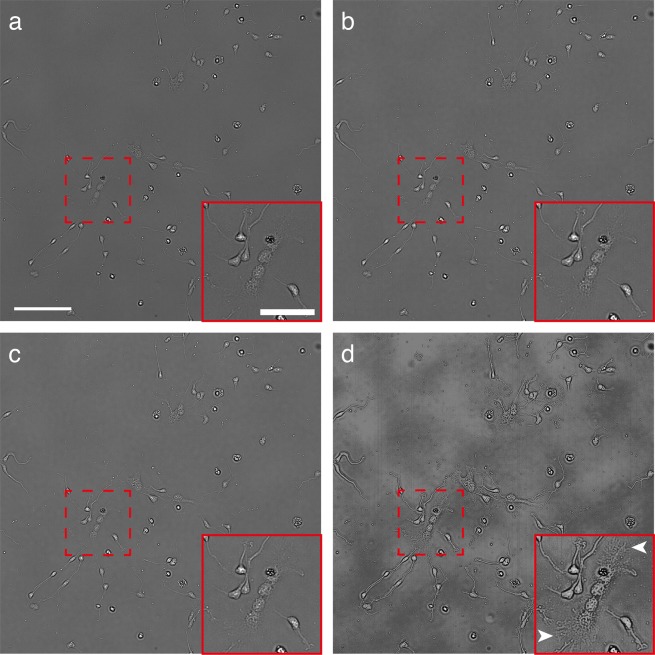
Hyperspectral images of differentiated NSCs. This figure shows spectral images obtained at 450-nm (**a**), 550-nm (**b**) and 650-nm wavelengths (**c**). The cluster image was constructed from 25 spectral images (**d**). Arrowheads: Edges of the expanding cell bodies. Scale bar: 100 μm, 50 μm (in insets).

Using the HSI microscopy with the settings mentioned above, we observed differentiated NSCs, which consisted of neurons and astroglia (glia) cells (Fig. 2). Using transmission images with 25 bands, including the wavelengths of 450 nm (Fig. 2a), 550 nm (Fig. 2b) and 650 nm (Fig. 2c), a cluster image was constructed (Fig. 2d, see the ‘Flat-field correction and cluster image’ subsection in the Methods section for clustering method). As shown in Fig. 2, details in the cellular structures (e.g. edges of the expanding cell body, Fig. 2d arrowheads) could be observed in the HSI cluster images (see Supplementary Fig. S4 for a comparison with bright field and phase contrast images).

### Class assignment and signal analysis

**Figure 3:**
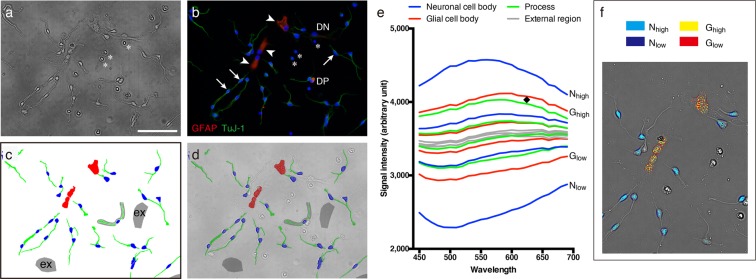
Cellular components and spectral analysis. This figure shows a cluster image of NSCs (**a**) and the corresponding immunofluorescent image (**b**: TuJ-1 in green, GFAP in red, nuclei in blue). (**c**) Pixel-wise class assignments: Neuronal cell bodies (blue), glial cell bodies (red), processes (green) and the external region (gray). (**d**) Superimposed image of (**a**,**c**). (**e**) Major average pixel-wise spectra of four object classes. (**f**) Pixel-wise spectra map for neuronal cell body and glial cell body corresponding to (**e**). Most of the objects belonging to each class contained all four major spectra, and each spectrum corresponded to a different intracellular location. In the neuronal cell bodies, the center areas had a high intensity spectrum N_high_, on the other hand, the edges of the cells showed a low intensity spectrum N_low_ (**f**). In the glial cell bodies, a high intensity spectrum G_high_ was observed only in the center areas, whereas a low intensity spectrum G_low_ was observed broadly in the edges of the cells and the inner areas (**f**). Arrows: Neurons. Arrowheads: Glia cells. DP: Double positive cells. DN: Double negative cells. Asterisks: Excluded cells. Black diamond: Wavelength specific differences between spectra. N_high_ and N_low_: High and low intensity major spectra of neuronal cell body. G_high_ and G_low_: High and low intensity major spectra of glial cell body. Scale bar: 100 μm.

In order to achieve a detail analysis and cell classification evaluation, which is mentioned below, we assigned cellular components into four classes, including the neuronal cell body, the glial cell body, the process of both types of cells and the external region, referring to immunostaining against a neuronal marker (neuron-specific β-III tubulin, TuJ-1) and a glial marker (glial fibrillary acidic protein, GFAP) (Fig. 3). We only used TuJ-1 single positive cells (Fig. 3b, arrows) as neurons and GFAP single positive cells (Fig. 3b, arrowheads) as glia, and excluded double positive cells (Fig. 3b, DP), double negative cells (Fig. 3b, DN) and cells which had a small nuclei and no cytoplasm or processes (Fig. 3a and b, asterisks). The cell bodies of each cell type were assigned independent classes (Fig. 3c, blue area: neuronal cell, red area: glial cell), but the processes of both cell types were combined into one class (Fig. 3c, green area). The external regions were included in order to obtain better segregation between the cells and the external area (Fig. 3c, gray area, also indicated as ex).

Figure 3e shows four major average pixel-wise spectra for each class in an acquired image which contained 36 neuronal cell bodies (total of 45,460 pixels), 4 glial cell bodies (total of 26,650 pixels), 49 processes (total of 56,006 pixels) and 8 external regions (total of 131,718 pixels). As shown in Fig. 3f, most of the objects belonging to each class contained all four spectra, and each spectrum corresponded mainly to a different intracellular location. In the neuronal cell bodies, the center areas had the high intensity spectrum N_high_, on the other hand, the edge of cells showed the low intensity spectrum N_low_ (Fig. 3f, cyan and blue pixels). In the glial cell bodies, the high intensity spectrum G_high_ was observed only in the center areas, whereas the low intensity spectrum G_low_ was observed broadly at the edge of the cells and in the inner areas (Fig. 3f, yellow and red pixels). Notable intensity differences in the spectra were observed among the classes (Fig. 3e), for example, the neuronal cell bodies consisted of both brighter signals and darker signals, compared with the other classes, and the external regions consisted mainly of mean intensity signals (see Supplementary Fig. S5 for standard deviation of the spectra). In addition, some wavelength specific differences were also observed, for example, a spectrum of the glial cell body had higher intensities at longer wavelengths, compared with the spectrum of the process (Fig. 3e, black diamond symbol).

### Overview of classification procedure

**Figure 4:**
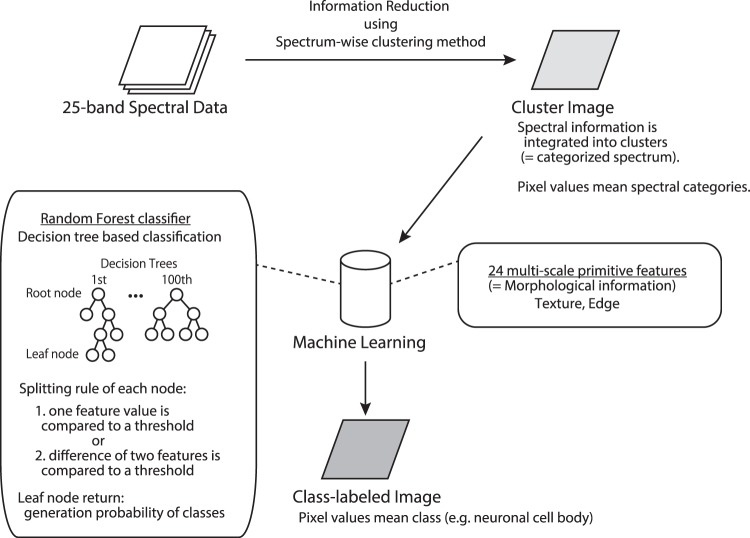
Overview of the cell classification procedure. 25-band spectral data was converted to the cluster image using a spectrum-wise clustering method. The pixel values of the cluster image indicate spectral categories. Pixel-wise cell classification was performed on the cluster image using in-house machine learning software, and the software returned the class-labeled image as a result. The machine learning software implemented a Random Forest classifier and multi-scale morphological features were employed.

Figure 4 shows an overview of the classification procedure. In general, raw hyperspectral data has too much information and redundancy, which interferes with the performance of machine learning systems. In order to overcome this demerit, we employed a clustering method for information reduction. First, 25-band spectral data was converted to a cluster image using a spectrum-wise clustering method (k-means algorithm, see the ‘Flat-field correction and cluster image’ subsection in the Methods section for detail). The pixel values of the cluster image indicate the spectral categories. Pixel-wise cell classification was performed on the cluster image using in-house machine learning software, and the software returned the class-labeled image as a result. Using categorized spectral information in the cluster image together with the morphological features, the system realized a comprehensive classification based on spectral and morphological characteristics.

### Cluster images

**Figure 5:**
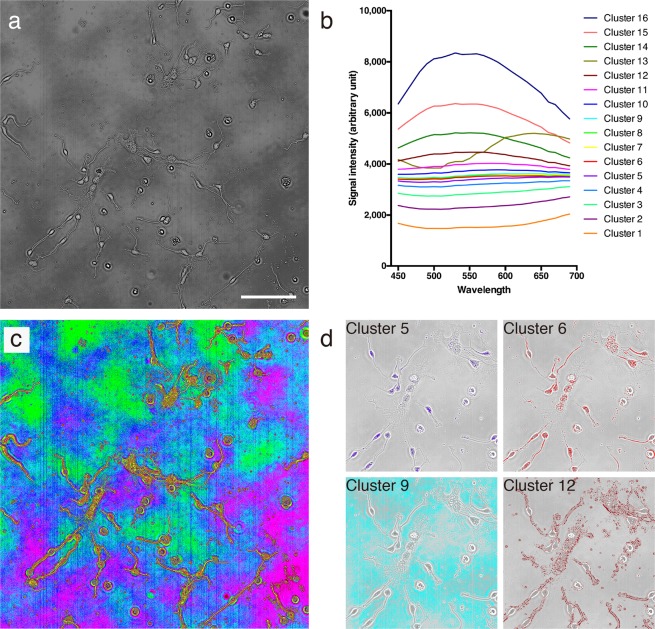
Cluster image. A cluster image of NSCs (**a**) and the average spectra of the clusters (**b**). Pixels were assigned to clusters based on 25-wavelength signals. (**c**) Color-coded cluster image of (**a**). (**d**) Single-colored cluster image. Colors in (**c**,**d**) correspond to the colors in panel (**b**).

Figure 5 shows the details of a cluster image constructed by the spectrum-wise clustering method. All of the pixels in the flat-field correction images were separated into clusters, based only on spectral similarity for information reduction (in this figure, the number of clusters was set to 16 for better visibility). Figure 5b shows the average spectra for clusters in a cluster image (Fig. 5a; see Supplementary Fig. S6 for standard deviation). As shown in the color-coded cluster image (Fig. 5c), different clusters were assigned almost exclusively to the cell region (yellow, red, dark purple) and the external region (green, cyan, blue, magenta). Among the 16 clusters, there were clusters which were only seen in neuronal cell bodies (Fig. 5d, cluster 5), clusters which were seen broadly in cells (Fig. 5d, cluster 6), clusters which were exclusively seen outside of cells (Fig. 5d, cluster 9) and clusters composed of the edges of cells and subcellular components (Fig. 5d, cluster 12). Even clusters which had close intensities each other were clearly assigned to different objects (Fig. 5b,d, cluster 6 and 9; where the average difference in signal intensity through the wavelengths was 74.0 ± 17.9 arbitrary unit, a.u.).

### Cell classification evaluation

**Figure 6:**
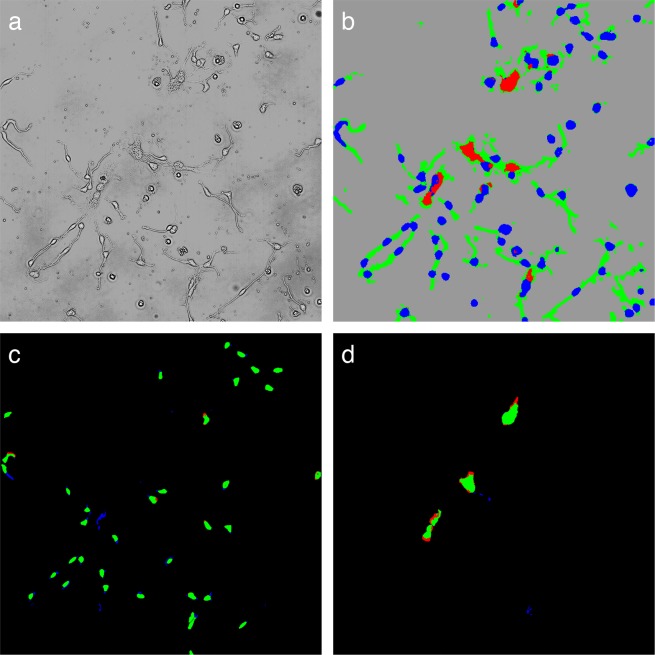
Cell classification. (**a**) Input image for classification (cluster image). (**b**) Classification result of the cluster image (**a**): Neuronal cell bodies in blue, glial cell bodies in red, processes in green, and the external region in gray. Single class result image for neuronal cell bodies (**c**) and glial cell bodies (**d**) True positives are shown in green, false positives in blue, and false negatives in red.

In differentiated NSCs, several types of cells exist, like neurons and glia cells, and the differentiation processes were dynamically affected by the surrounding environment. In order to evaluate the feasibility of monitoring live NSCs, pixel-wise classification on four object classes was performed using multiple culture dishes. In the evaluation of the classification procedure, with a total of 19 images, 18 images were used for building the clustering model (32 clusters) and for machine learning training. The last remaining image was clustered based on the above clustering model and classified using the trained machine learning system (see the Methods section for the details of the procedure). All 19 combinations were evaluated and the results were averaged. Figure 6 shows an example of a classification result. With a cluster image as an input of classification (Fig. 6a), the extracellular region was clearly classified against the cellular components (Fig. 6b, gray: external region, blue: neural cell body, red: glial cell body, green: process), and most of the pixels in the neuronal cell bodies and the glial cell bodies were correctly classified (Fig. 6c for neuronal cell body, Fig. 6d for glial cell body, green indicates pixels that were classified correctly). Some misclassifications between neuronal cell body and process (Fig. 6c, blue pixels), and between glial cell body and external region (Fig. 6d, red pixels) were observed at marginal regions.

Table 1 summarizes the results of the evaluation of the classification procedure. This evaluation included 378 neuronal cell bodies (total of 548,626 pixels), 39 glial cell bodies (total of 199,876 pixels) and 471 processes (total of 402,514 pixels). In the pixel-wise results, the average F_0.5_ score for these 3 classes was 0.801 ± 0.095 (mean ± s.d.). Most of the pixels in the neuronal cell bodies were correctly classified (recall = 0.943 ± 0.022). Although 43% of the pixels in the glial cell bodies were not classified properly, the predictions for this class were the most precise, among the 3 classes tested (precision = 0.891 ± 0.103). More than 99% of the extracellular pixels were correctly classified with high precision (99.4%). In the object-wise results, an object was considered as correctly classified when the number of correctly predicted pixels was highest among the respective pixels, the average F_0.5_ score for these 3 classes was 0.901 ± 0.172. All of the objects studied were classified precisely (minimum average precision was 0.904 ± 0.195), and a minimum of 72% of the objects studied (88% of the objects on the average) were recalled properly.

**Table 1:** Summary of the classification evaluation.

Pixel-wise result	# of pixels	Precision	Recall	F_0.5_	F_1_
Neuronal cell body	548,626	0.827 ± 0.085	0.943 ± 0.022	0.846 ± 0.071	0.878 ± 0.046
Glial cell body	199,876	0.891 ± 0.103	0.573 ± 0.216	0.776 ± 0.117	0.673 ± 0.168
Process	402,514	0.772 ± 0.098	0.829 ± 0.055	0.780 ± 0.077	0.794 ± 0.050
External region	14,329,240	0.994 ± 0.013	0.996 ± 0.003	0.995 ± 0.011	0.995 ± 0.008
Average (excluding external region)		0.830 ± 0.106	0.781 ± 0.201	0.801 ± 0.095	0.782 ± 0.133
**Object-wise result**	**# of objects**	**Precision**	**Recall**	**F**_**0**. **5**_	**F**_**1**_
Neuronal cell body	378	0.918 ± 0.051	0.992 ± 0.017	0.931 ± 0.043	0.953 ± 0.030
Glial cell body	39	0.904 ± 0.195	0.724 ± 0.326	0.797 ± 0.267	0.751 ± 0.279
Process	471	0.993 ± 0.021	0.917 ± 0.072	0.976 ± 0.022	0.952 ± 0.039
Average		0.938 ± 0.122	0.878 ± 0.221	0.901 ± 0.172	0.885 ± 0.187

All values (except total numbers of pixels/objects) are shown as an average of 19 tests ± s.d.

## Discussion

Considering the growing demand for a viable label-free observation method in the biomedical field, we investigated the feasibility of using the HSI method for rapid label-free observation in stem cell research at a practical level. The proposed HSI microscopy employed in this study was simply composed of a research grade microscope, a liquid crystal tunable bandpass filter and an industry grade area scan camera. Using our HSI microscopy method, with a 562 × 562 μm^2^ field of view, a 2048 × 2048 pixel image containing 63-wavelength spectra can be obtained in 30 seconds (even half of the data were not implicated in the following cell classification), which is far more rapid than RS and the laser scanning confocal HSI microscopy method. Selecting signal wavelengths one by one using the tunable filter, you can compensate for optical losses and wavelength differences in quantum efficiency to some degree, which originates in various parts of the apparatus, by controlling the exposure time. Hence our HSI microscopy system has a uniform signal dynamic range across a wide range of wavelengths (for the wavelengths included in the analyses, the number of intensity values contained in a raw object image was 2,414 ± 108, Supplementary Fig. S8). In regard to the reference spectra, no strict reference was required in the proposed HSI microscopy, and so spectra of the illumination without specimens were used as a flat-field reference. We evaluated the feasibility of using other specimens without cells (only culture dishes and media) as a reference, however, the intrinsic variations (for example, the thickness/flatness of the glass, or the depth of the medium) and the differences of these parameters between the reference and target specimens resulted in less stable measurements. The major downside to using our HSI microscopy is its quantitative capability. With a reference spectra which is acquired without a specimen, the spectra obtained consists not only of cell specific information, but also components that stem from culture dishes and media. Therefore, it is expected that there will be some difficulty in directly evaluating the exact cell state, based only on the acquired raw spectra. Another factor which affects the quantitative capability is chromatic aberration. Considering the working distance, the correction collar for compensating for cover glass thickness and the transmission performance across the wavelengths we focused on, we chose a universal semi-apocromatic objective lens in this study (refer to the Methods section for the details). In contrast to the previous confocal HSI microscopy, which implemented a zero chromatic aberration reflective objective lens^20^, due to the remaining chromatic aberration, the focal plane for each wavelength is slightly shifted individually. Consequently, a light beam transmitted through one point of the object affects pixels near the corresponding object location in the acquired image depending on the wavelength. Nonetheless, as shown in the classification evaluation in this study (Fig. 6 and Table 1), the spectra acquired by our HSI microscopy could be successfully utilized for classifying cell types under certain circumstances (using the same product dishes and media, and cells of the same lineage). Taking the results of the cell classification evaluation into account, our microscopy method seems to retain enough quantitative accuracy for some practical applications in stem cell research.

Recent studies have reported differences in the refractive index (RI) among subcellular components, i.e., nuclei have a lower refractive index than cytoplasm^29^, and the mitochondria, lysosomes and endoplasmic reticulum have their own unique RIs^30–32^, respectively. In addition to intracellular RI differences, cell type dependent differences^33,34^ and cell state dependent differences^34,35^ have also been demonstrated. In the observation of transparent material using reflectance hyperspectral/multispectral microscopy, the backscattered light is imaged, which relies on the inherent differences in the RI of structures (the cytoplasm is shown as a bright intensity and the nucleus scatters less light, so it is shown as a dark area)^20,25,26,36,37^. On the other hand, it is considered that our HSI microscopy, with its implemented, transmitted light path, relies on chromatic dispersion (the dependency of the RI on the wavelength). Transmitted light passing through subcellular components causes refraction due to the differences in the RIs of those subcellular components. Because the angle of refraction is dependent on the angle of incidence, the RIs of the subcellular components, which have different shapes and densities, cause refraction that varies differently from each other, accompanied by wavelength differences. Using the proposed HSI microscopy presented in this study, the integrated results of these refractions could be recorded for each wavelength.

When you assess the feasibility of adapting some method for a certain application, the materials and experiments you use, and the evaluation scheme employed are highly important. Regarding materials and experiments, we focused on the study on developmental brain disease using NSCs in this study. In malformed brains, which are observed in some developmental brain diseases (for example, Down syndrome), impaired differentiation has been reported, resulting in an altered ratio of neurons to glia^38^ and abnormal astrocyte reactivity^39^. For this reason, we conducted a cell classification evaluation with differentiated NSCs, which were, as previously reported^4^, differentiated into TuJ-1 positive neurons and GFAP positive glia (Fig. 3). Regarding the evaluation scheme, evaluating practical performance is important. In previous studies, the authors manually extracted the target spectra in cells from the data obtained, and evaluated classification performance only with extracted spectra^25,26^. This evaluation scheme is useful for evaluating feasibility at the conceptual level, but the lack of an evaluation for the extraction of the target spectra may cause performance discrepancy in practical applications. In this study, focusing on processing spectra and image analysis, we employed a pixel-wise classification based on machine learning, and included almost all types of pixels into the evaluation (except pixels consisting of double positive and double negative cells). Using this evaluation scheme, you can tell simultaneously how spectra are precisely classified between the spectra of interest and non-interest spectra (like extracellular regions), and between the spectra of interest (for example, between neurons and glia). Hence, we concluded that this evaluation scheme is more practical than those previously utilized. In this study, by means of the pixel-wise classification evaluated with this method, differentiated NSCs were precisely classified (an average of 88% of the objects of interest were correctly classified with a precision average of 94%, and more than 99% of the extracellular pixels were correctly segregated). We consider that this performance is promising for some experiments concerning developmental brain diseases. For example, in a study on Down syndrome, the number of neurons decreased 30–40% in the patient group^38^. In this case, you can correctly detect the deference between cells of the patients and cells of the control group with our classification system (with 12% misclassification). In the evaluation results, the recall values (indicating how many relevant pixels/objects were properly predicted) of the glial cell bodies were lower than that for the other classes. This was one of the reasons that the contrasts in the widely expanded glial cells were rather low, especially in regions distal from the nuclei. As mentioned above, the downside of our microscopy method is its relatively poor quantitative capability. Refining the reference spectra or the achromatic conditions would improve these contrasts.

The main limitation of this study was the cell types (TuJ-1 and GFAP double positive and double negative cells) that were excluded from the classification evaluation. In this study, some cells showed intermediate level signals of both TuJ-1 and GFAP, some showed definitely negative against both antibodies, and yet other cells showed a strongly positive signal for one staining, while showing an intermediate signal for another. These cells were excluded because we did not characterize these cells in detail. However, because these cells, which might be undifferentiated stem cells, play important roles in some cases, we consider that further studies should be conducted in order to characterize these cells and evaluate them for classification performance. Another limitation was that we stained cells before image acquisition. We took this approach to obtain ideal pixel-wise ground truth information (answer labels) for training of machine learning system and the evaluation of the classification performance, but the staining process might have affected the statuses of the cellular components, e.g. RIs and morphology, resulting in artificial signals. Further studies using living cell acquisition should be performed to confirm advantages of proposed HSI microscopy.

In summary, we investigated the feasibility of a label-free HSI microscopy method combined with machine learning for application in stem cell research. With the sole use of commercially available apparatus, we developed a high speed HSI microscopy method capable of wide area observation. Employing a pixel-wise machine learning classification method on data obtained using the HSI microscopy method, we demonstrated that differentiated NSCs were precisely classified in a practical evaluation. The results indicate that the proposed HSI microscopy observation method is feasible for label-free observation in stem cell research.

## Methods

### Apparatus

An inverted microscope IX73 (Olympus, Tokyo, Japan) was utilized in this study. The following units were also installed on the microscope, a halogen lamp housing U-LH100L-3 without any IR cut filter (Olympus), a diffuser 45FR (Olympus), a condenser unit IX2-LWUCD (NA = 0.55, Olympus), and a semi-apochromatic objective lens LUCPLFLN 20× (Olympus). A liquid crystal tunable bandpass filter Kurios-WB1 (Thorlabs, Inc., Newton, NJ, USA) was inserted between the left side port of the microscope and an area scan camera acA2040–25 gm, with a 1 inch sensor, 2048 × 2048 pixels, 12 bit-depth monochrome (Basler AG, Ahrensburg, Germany). Two adapters were inserted in front of and behind the tunable bandpass filter. In the front adapter, a shortpass filter was installed (FESH0750, Thorlabs, Inc., with a cut-off wavelength of 750 nm).

The bandwidth of the tunable bandpass filter was: 18 nm full width at half maximum (FWHM) at the 450-nm wavelength, 31 nm at the 550-nm wavelength and 43 nm at the 650-nm wavelength. The minimum incremental step size was 1 nm and tuning accuracy was ± FWHM/10. Detailed information can be obtained from the maker’s web site^40,41^.

All methods were performed in accordance with the relevant guidelines and regulations by the Independent Ethics Committee at Kyoto Prefectural University of Medicine.

### Acquiring hyperspectral images (HSI)

In order to acquire the hyperspectral images, the microscopic parameters were set as follows. The light intensity was set to the maximum, the field iris diaphragm was fully opened, and the aperture iris diaphragm was set to the minimum position (the numerical aperture of the illumination was 0.063). The condenser height was adjusted for the specimen observation, while the objective correction collar was adjusted to get the sharpest images at a 550-nm wavelength (Supplementary Figs S1 and S2). The camera parameters were set as follows. Gain was set to 36 (lowest), the black level was set to 0, and gamma was set to 1.0, with no binning. Exposure times were adjusted depending on the wavelength (Supplementary Fig. S7). The field of view was 562 × 562 μm^2^. The camera and the tunable bandpass filter were synchronously controlled by in-house software, and from a 420-nm to 730-nm center wavelength images (5-nm pitch, 63 images) were acquired sequentially. Exposure times were at most 130 ms at 420-nm wavelength and wavelength switching time between 5-nm adjacent frames were less than 5 ms. The total acquisition time was 30 s (including extra time for assuring wavelength switching and data transfer). Dark images (with light path shuttered) and flat-field reference images (illumination image without specimens) for image correction were acquired with the same exposure times as raw object images, depending on the wavelength.

### Flat-field correction and cluster image

The images with flat-field correction (FFC image) were calculated from a raw object image (O), a dark image (D) and a flat-field reference image (F), as shown in the following equation:

$$FFC=(O-D)/(F-D)$$

Based on 25-band signals from a 450-nm to 690-nm wavelength at a 10-nm pitch, all of the pixels in the FFC image were clustered into 16–64 clusters, then new pixel values were assigned considering the cluster’s average signal intensity among the 25 bands (cluster image). The clustering model was built by k-means algorithm with the k-mean^++^ seeding method^42^ implemented in scikit-learn, a machine learning module for the Python language^43^ (version 0.17.1, http://scikit-learn.org). The details of the k-means algorithm are explained elsewhere^44^. Briefly, after initial centroids were chosen, each sample was assigned to the nearest centroid that formed initial clusters. The Euclidean distance was calculated with the 25 spectral values used in this study. Then new centroids were computed by taking the average value of all of the samples belonging to each cluster. The clusters were updated as all samples were re-assigned to their nearest centroids. The clusters were updated 30 times in this study. The ‘predict’ function was used to cluster the pixels in the FCC image, which returns the nearest cluster id based on a previously built clustering model.

### Cell classification procedure

The machine learning software implemented Random Forest^45^ as a classifier utilizing multi-scale morphological (texture and edge-based) features as follows: The Hessian, the Laplacian, the standard deviation and the circular standard deviation^46^. The kernel sizes were set to 7 pixels for the calculation of these features, and three scaling factors 1, 1/2 and 1/4 were used (in other words, half size and quarter size image as well as original size cluster image were used to extract features). A total of 24 features were included for cell classification.

Random Forest is a kind of decision tree classifier. In our implementation, the classifier consisted of 100 decision trees; each of which had a maximum of 50-depth nodes. Each node had its own splitting rule, which was ether one of the following two types: one feature value was compared to a threshold, or the difference of two feature values was compared to a threshold. The leaf nodes had generation probabilities of classes.

In the training phase, the trees grew from the root node, node by node using a training dataset. Around 400 candidate nodes that had randomly chosen rules (combination of type, features and threshold) were evaluated based on the entropy reduction, and the best node was added to the tree as a child node. Growth of the tree was stopped when any candidates could not reduce the entropy. Then the leaf node was created and the generation probability of the classes was calculated based on the remaining training data. Different sets of randomly sampled training data were assigned for different tree training.

In the classification phase, all of the pixels in the test image were evaluated from the root node in the trees of the trained classifier, which went down to the left or right according to the splitting rules, and reached the leaf node. The generation probability of classes in the leaf node was served as the result of the tree. After averaging all of the generation probabilities of 100 trees, the class which had the highest probability was returned as the classification result for the pixel.

### Materials for cell classification evaluation

The experiments using NSCs were approved by the Independent Ethics Committee at Kyoto Prefectural University of Medicine in accordance with relevant guidelines and regulations after obtaining informed consent from legal guardians. Human neural stem cells from a normal post-mortem brain^4^ (donor n = 1) were seeded on 0.01% poly-L-ornithine (P4957-50ML, Sigma–Aldrich Co., LLC.) coated 3.5-mm glass bottom dishes (D11530H, Matsunami Glass Ind., Ltd.) at a density of 3.0 × 10^5^ cells per dish, then differentiated for 7–9 days with Neurobasal medium (21103–049, Thermo Fisher Scientific, Inc., MA, USA) containing 2% MACS NeuroBrew-21 (Miltenyi Biotec, Bergisch Gladbach, Germany), 2 mM L-Alanyl-L-glutamine (04260–64, Nacalai Tesque) and 1% penicillin-streptomycin. Three dishes were prepared for the evaluation, the passage numbers of 2 dishes were the same, and the other was subcultured once more.

Next, the cells were stained with 3.6 μM Hoechst 33342 solution (346–07951, DOJINDO LABORATORIES, Kumamoto, Japan) for 10 min at 37 °C, and fixed with 4% paraformaldehyde for 10 min at room temperature (23 °C). After blocking with 5% BSA (A9647-10G, Sigma–Aldrich Co., LLC.) and 0.1% Triton-X 100 in PBS (−), the cells were incubated in the following primary antibodies overnight at 4 °C: Neuron-specific β-III tubulin (TuJ-1, mouse monoclonal, MAB1195, R&D Systems, Minneapolis, MN, USA, 1:300) and glial fibrillary acidic protein (GFAP, rabbit polyclonal, 23935-1-AP, Proteintech, Rosemont, IL, USA, 1:300). Finally, the cells were incubated in the following secondary antibodies for 1 h at room temperature (23 °C): Goat anti-mouse IgG (Alexa Fluor 488, A-11029, Thermo Fisher Scientific, Inc.) and goat anti-rabbit IgG (Alexa Flor 546, A-11071, Thermo Fisher Scientific, Inc.).

### Cell classification evaluation

A total of 19 images were acquired across 3 dishes. All of the images included at least one TuJ-1 positive cell and one GFAP positive cell. Four classes were designated for evaluation: 1. TuJ-1 positive cell body, 2. GFAP positive cell body, 3. Process of either cell type and 4. External region of a cell. TuJ-1 and GFAP double positive, as well as double negative cells were excluded. Leave-one-out (LOO) style cross validation was performed to estimate classification performance. In this study, one image was selected for test data, and the clustering model and classification model were trained with the remaining 18 images. Then we clustered and classified the test data. For the performance indices, precision (how many selected items are relevant), recall (how many relevant items are selected) and the F_β_ score (β = 0.5 and 1) were calculated by averaging the results of all 19 combinations. The F_β_ score is defined as shown below:

$${F}_{\beta }=(1+{\beta }^{2})(PRE\ast REC)/({\beta }^{2}\ast PRE+REC)$$

where PRE is the precision value and REC is the recall value. The F_1_ score is also known as Dice’s coefficient.

## Supplementary information

Dataset 1
