# Supplementary material for: Label-free classification of neurons and glia in neural stem cell cultures using a hyperspectral imaging microscopy combined with machine learning
Source: Sci Rep. 2019 Jan 24;9:633. doi: 10.1038/s41598-018-37241-y (PMC6345994; doi:10.1038/s41598-018-37241-y)
Supplement: Supplementary file 1 — Dataset 1 [file 41598_2018_37241_MOESM1_ESM.pdf]

Label-free classification of neurons and glia in neural stem cell cultures using a hyperspectral imaging microscopy combined with machine learning

Hiroshi Ogi[1,2], Sanzo Moriwaki[1,3], Masahiko Kokubo[3], Yuichiro Hikida[3], Kyoko Itoh[1,2,*]

1. Department of Interdisciplinary Research & Development, Graduate School of Medical Science, Kyoto Prefectural University of Medicine (KPUM), Kyoto, Japan
2. Department of Pathology and Applied Neurobiology, Graduate School of Medical Science, Kyoto Prefectural University of Medicine, Kyoto, Japan
3. SCREEN Holdings Co., Ltd.

Hiroshi Ogi and Sanzo Moriwaki contributed equally to this study

*** Corresponding author:**
Kyoko ITOH, M.D., Ph.D.
Department of Pathology and Applied Neurobiology**,** Graduate School of Medical Science, Kyoto Prefectural University of Medicine**,**
465 Kajii-cho, Kawaramachi-Hirokoji, Kamigyo-ku, Kyoto, 602-8566 Japan.
Fax number: +81-75-251-5849.
Telephone number: +81-75-251-5849.
E-mail: kxi14@koto.kpu-m.ac.jp

Figures

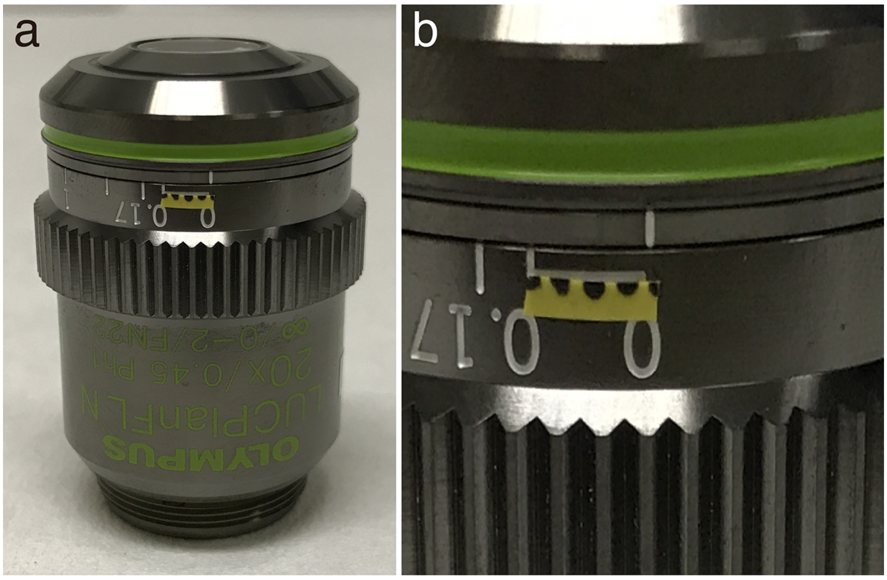

Figure S1. Correction collar configuration. The objective lens with the correction collar at a minus position was used for acquiring the hyperspectral images (a). (b) Magnified view of (a).

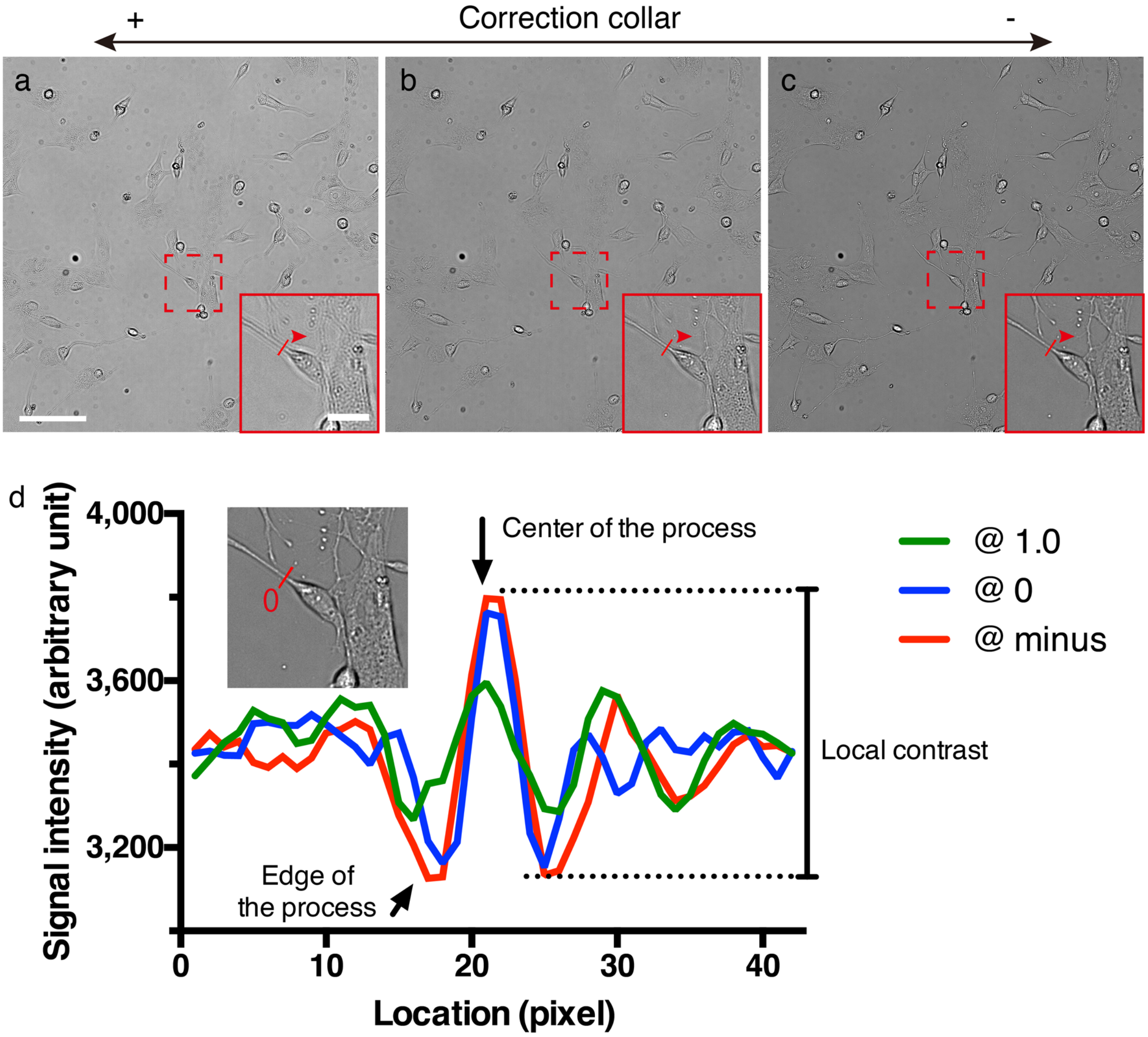

Figure S2. Correction collar configuration for the HSI acquisition. In-focus images were acquired at a 550-nm wavelength with different correction collar configurations (a-c). The correction collar was set at 1.0-mm (a), 0-mm (b) and a minus position (c). The images were sharpest at the minus position, and a small process (red arrow head in insets) was recognized only with the minus configuration (c). The global contrast values, defined as the maximum difference of intensity in the image, were 7,313 (arbitrary unit, a.u.) at the 1.0-mm position collection collar, 8,131 a.u. at the 0-mm position and 10,153 a.u. at the minus position. The local contrast was defined as the difference between the highest intensity at the center of the process and the lowest intensity at the edges of the process in the line profile (d). The local contrast values were 318.9 a.u. at the 1.0-mm position, 605.1 a.u. at the 0-mm position and 667.0 a.u. at the minus position. Insets: Magnified view of the dashed rectangle areas. The '0' in the inset in (d) indicates location 0 at x-axis in the line profile. @1.0: 1.0-mm position, @0: 0-mm position, @minus: the minus position. Scale bar: 100 µm, 25 µm (in insets).

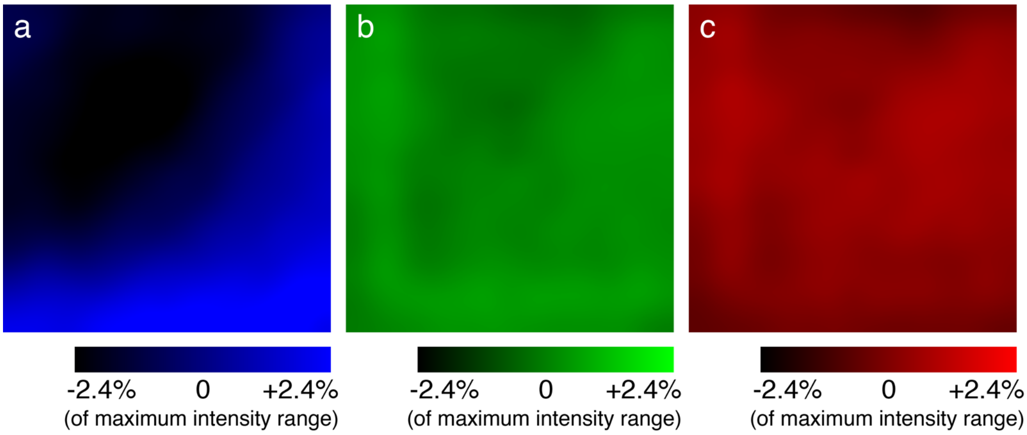

Figure S3. Spatial stability of the optical setup. The figure shows the non-uniformity of the intensity at the 450-nm (a), 550-nm (b) and 650-nm wavelengths (c). The range of the figure indicates the percentage of the maximum intensity range (=4,096).

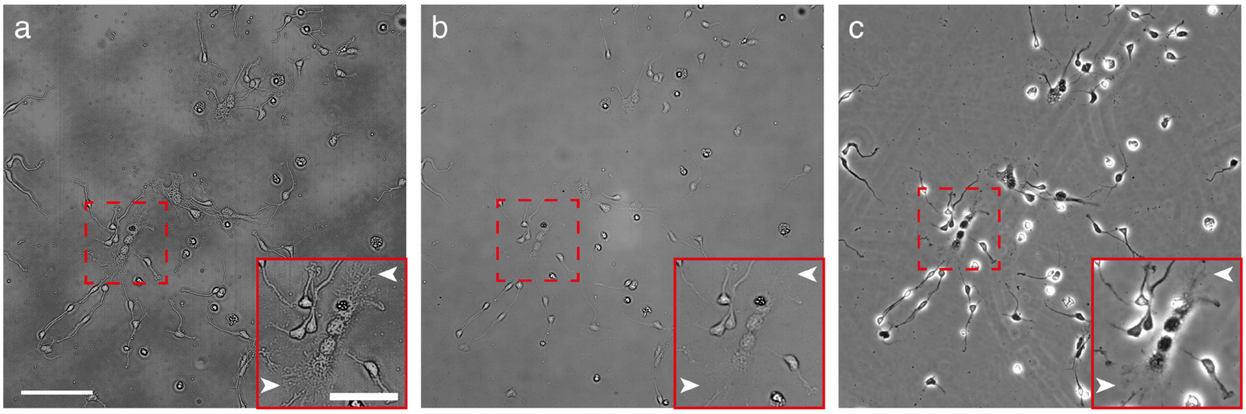

Figure S4. Comparison between HSI, bright field and phase contrast images. Edges of the expanding cell body (arrowheads) could be observed in the HSI cluster image (a), compared with bright field image (b), without any destructive artifacts for image analysis, like the halo artifact often seen in phase contrast image (c).

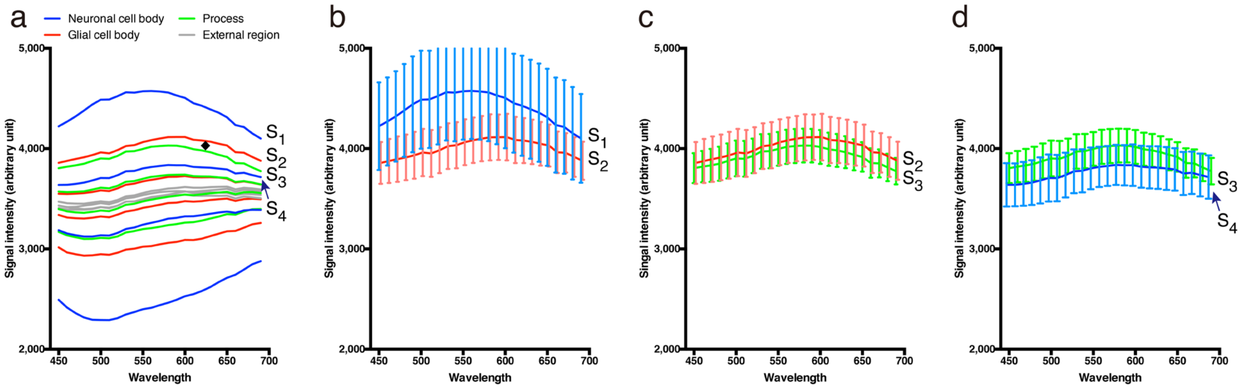

Figure S5. Major spectra in the object classes. Major average pixel-wise spectra of four object classes (a). A plot of the highest spectrum of the neuronal cell body (S_1) and the highest spectrum

of the glial cell body (S_2) with standard deviation (b). A plot of the highest spectrum of the glial cell body (S_2) and the highest spectrum of the process (S_3) with standard deviation (c). A plot of the highest spectrum of the process (S_3) and the 2nd highest spectrum of the neuronal cell body (S_4) with standard deviation (d).

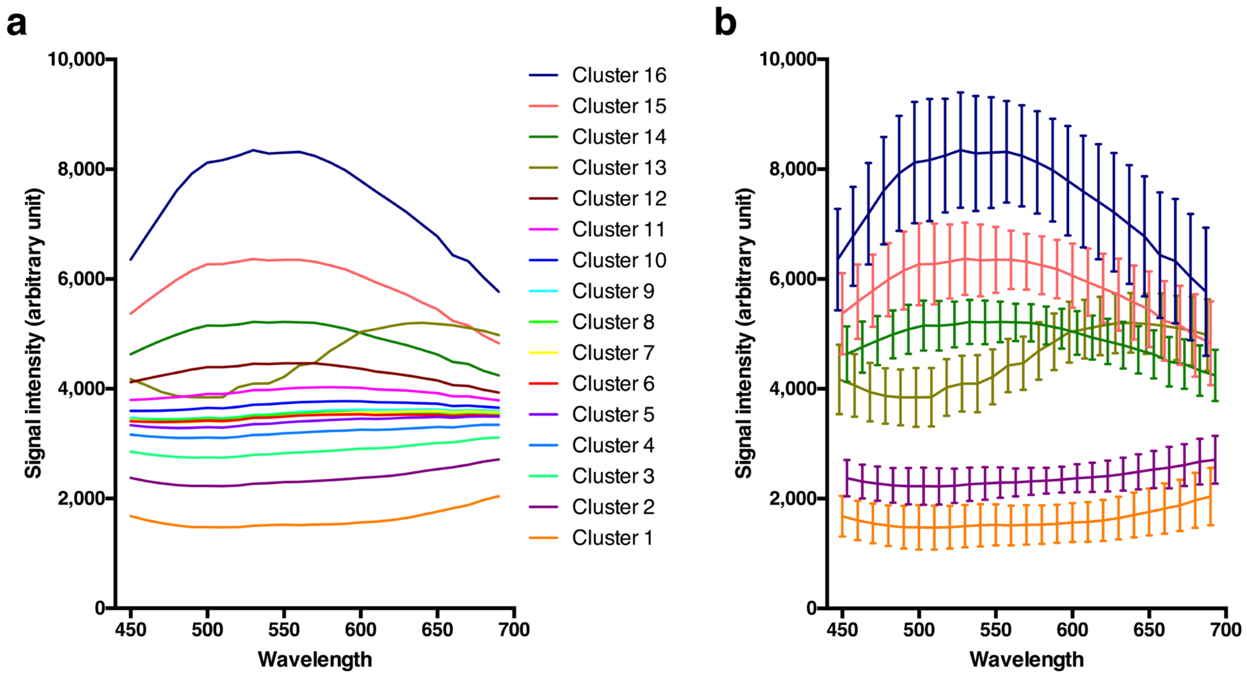

Figure S6. Average spectra of clusters in an HSI cluster image of NSCs. Six spectra plotted with standard deviation (b).

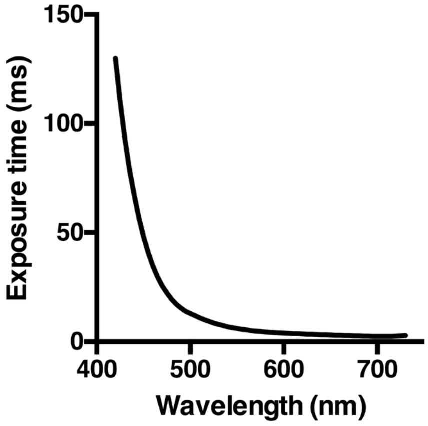

Figure S7. Exposure times. Exposure times were adjusted depending on the wavelength.

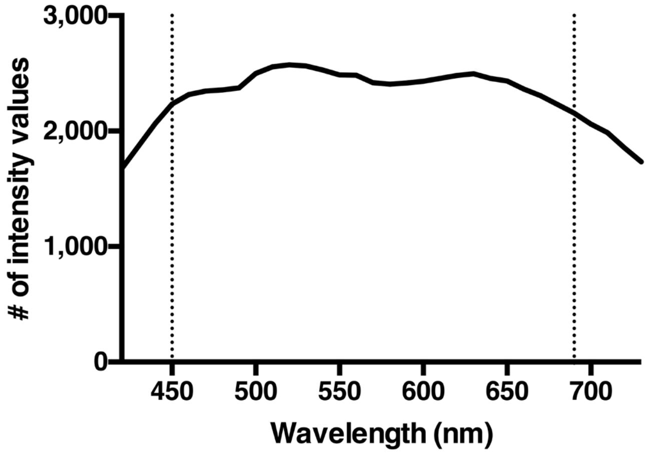

Figure S8. The number of the intensity values contained in a raw object image. For the wavelengths used in the analyses (between the dashed lines), the average number of the intensity values was 2,414 and the standard deviation was 108. The camera bit-depth was 12 bits, so the maximum number for these values was 4,096.
